# Ecosystem Services, Global Diversity, and Rate of Stonefly Species Descriptions (Insecta: Plecoptera)

**DOI:** 10.3390/insects10040099

**Published:** 2019-04-06

**Authors:** R. Edward DeWalt, Geoffrey Donald Ower

**Affiliations:** University of Illinois, Illinois Natural History Survey, 1816 South Oak St., Champaign, IL 61820, USA

**Keywords:** Plecoptera, biodiversity informatics, global species diversity, species discovery curves

## Abstract

Stoneflies (Insecta: Plecoptera) provide ecosystem services as indicators of water quality, as food for predators, as mediators of energy flow and nutrient cycling, and through cultural services related to recreation and artistic creativity. The Plecoptera Species File (PSF) aggregates stonefly nomenclature, distribution, and literature to help society and scientists understand the value of services stoneflies provide. Using PSF data, we examined global and regional diversity, compared species description rates, and predicted future species description numbers through the year 2100. Through 2018, extant species totaled 3718 with Temperate Asia having the greatest regional diversity at 1178 species. The Perlidae was the most species-rich of the 16 families at 1120 species. The recent global rate of species description was 43.6 species/yr, with Temperate Asia having the highest regional rate at 13.7 species/yr, followed by China and South America adding approximately 9.0 species/yr. We predicted that 1140 ± 130 new species would be described globally by 2050, and 2130 ± 330 by the year 2100, most of the increase occurring in China and South America. We discuss the possibility of reaching these predicted values.

## 1. Introduction

Stoneflies (Plecoptera) are an ancient order of insects whose fossil record extends 300 million years ago to the Pennsylvanian [1]. A combination of external morphological characters distinguish stoneflies from other insect orders: Most adults have two pairs of wings, larvae have two multi-segmented cerci, and the tarsus is three segmented with paired claws. The biology of stoneflies was recently reviewed by DeWalt et al. [2].

Using the definition of Bybee et al. [3], stoneflies exhibit a hemimetabolous metamorphosis consisting of egg, nymph or naiad (their preferred term), and adult life stages. Most stonefly researchers use nymph for the immature stage of stoneflies, we retain its use here. The aquatic nymphs grow gradually then transform to usually winged adults. Nymphs feed on decaying leaves and wood, encrusting algae, or on other invertebrates, and some species are known to undergo ontogenetic diet shifts [4]. Stoneflies utilize all stream sizes, inhabit high latitude or elevation lakes, endure a wide range of thermal regimes, and have evolved to complete their life cycles under a broad range of stream permanence conditions [2]. Adults are almost exclusively terrestrial, and approximately half of the species feed during this stage to support maturation of eggs [5,6]. Exceptions to nymphal and adult habitat use occur, for instance, nymphs of *Vesicaperla* McLellan, 1967 (Gripopterygidae) are terrestrial in New Zealand [7], and adults of *Capnia lacustra* Jewett, 1965 (Capniidae) never leave the depths of Lake Tahoe in California and Nevada, USA [8].

The highest species richness of stoneflies occurs in cool and coldwater streams draining mountains of temperate latitudes, but considerable diversity occurs in high-quality, warm-water streams. Species reside on every continent except Antarctica and avoid areas of continuous ice cover and vast deserts [9].

The objectives of this paper include the following:

1. Summarize existing ecosystem services of Plecoptera
2. Summarize global and regional species richness across large geographic regions
3. Examine the rate of species description globally and within large geographic regions
4. Predict the number of new species described globally and for two fast-growing regions for the years 2050 and 2100.

## 2. Ecosystem Services Provided by Plecoptera

Ecosystem services generally consist of four categories: provisioning, regulating, habitat, and cultural services [10]. Stoneflies provide services that meet at least three of these categories. Provisioning services from stoneflies, as a direct source of food for humans, is uncommon [11]. A few cultures in Asia and Africa eat stoneflies, but they likely do not occur in large enough numbers to be a dependable food source in urban areas. Stoneflies are also not easily reared in sufficient numbers to make them commercially viable [2]. They are, however, an important source of food for wild fish [12]. Stoneflies are also prey for other invertebrate and vertebrate predators in both the aquatic and terrestrial setting. They are one of a few insect orders to provide terrestrial prey in nearly every month of the year, with winter emergence of adults from a few families.

Stoneflies perform many regulating services. These include their use as indicators of water quality [13] and as mediators of nutrient cycling and energy flow across the land–water interface. They are often the first insects to be lost from freshwater systems after relatively minor nutrient enrichment, degradation of habitat or changes in aquatic thermal regimes [12]. Stoneflies are among the most pollution sensitive of aquatic insects, and yet exhibit a sufficiently broad range of sensitivity that a small contingent of species may exist in even moderately polluted streams [14]. 

This sensitivity leads to the imperilment of many stonefly species [15,16]. Plecoptera researchers use museum specimens to establish historical assemblages for comparison with contemporary fauna. Currently, there are over 150,000 verifiable, digitized specimen-based occurrence records in museums of the developed world [17]. Many more specimens remain uninvestigated in museums. Using these data researchers have documented extinctions (Illinois, USA: *Isoperla conspicua* Frison, 1935; *Alloperla roberti* Surdick, 1981) [15], regional extirpations [15,18], massive range losses [19,20], and declines of entire guilds of stoneflies (predators and long-lived species) [15]. Myriad reasons exist for these losses including acid precipitation [19], large scale hydrological modifications (channelization, tiling of fields, and riparian tree removal) [15], eutrophication [20], and sedimentation resulting from agricultural practices and regional urbanization [15]. One result of these losses is that smaller species with shorter life cycles have replaced long-lived species with direct egg hatching [15]. These losses are broad enough that entire generations of water quality biologists no longer recognize that multiple species of large, long-lived, predatory stoneflies were once common in streams, resulting in shifted regional baseline expectations (e.g., the shifting baseline syndrome of Soga and Gaston [21]). Most of the losses to date have not been climate related, though some climate impacts may already be occurring [22,23] and more severe consequences are predicted [24].

The ecological consequences of these stonefly species losses are not well understood, but others have hypothesized that it could upset the normal flow of nutrients and energy to downstream areas [25]. Of the 16 stonefly families, 11 are shredders of coarse organic matter and comprise a majority of the species in the order [9]. Loss of shredder stoneflies from headwaters due to acid precipitation [19], climate change [22], or pesticide application [26] could dramatically diminish the availability of fine organic matter causing a cascade of losses of downstream invertebrate and vertebrate species [27] that provide many other ecosystem services.

Cultural services provided by stoneflies are mostly recreational, involving trout fishing. Fishermen model both dry and wet artificial flies after stonefly nymphs and adults. They often examine the size and coloration of species available streamside to more accurately match the locally available nymphs and adults. Leiser and Boyle [28] wrote the definitive book on stoneflies for anglers, supported by illustrations, life history descriptions, and patterns for tying of artificial flies that mimic stoneflies. Trout anglers have also invested much time and funds into their hobby, purchasing licenses and gear, producing videos and workshops, shooting still photographs of live insects, and creating physical artwork. They are often politically involved advocates for protecting streams where both stoneflies and trout reside.

## 3. Compilation of Data on Global Diversity of Plecoptera

Basic biological information is necessary to help society and scientists understand and measure the value of the ecosystem services that stoneflies provide. Taxonomic catalogs enhance our basic knowledge by compiling inventories of taxon names, type information, classification, distribution data, and links to scientific literature. Until recently, these catalogs were hard copy snapshots-in-time that have been difficult to summarize and update. The Plecoptera Species File is built on the foundation of past catalogs but is published on an updatable, open electronic platform [9].

The nomenclature of stoneflies has been cataloged several times in the 20th century. The first major effort was completed by Claassen [29] and totaled just under 1000 species. His work presented little more than a list of valid species, their synonyms, literature references, and distribution. The next was the Herculean effort by Illies [30] who provided an updated classification system, biogeographical analyses, reclassification of many of the subgenera to generic status, and listed approximately 1600 valid species and their synonyms. He also recorded type species of genera, type information for species, taxonomic literature, expansive distributional information, and a list of species names whose status was uncertain at the time. Although Illies compiled a large amount of valuable information, many valid species names and references were not included, which prompted Zwick [31] to publish an update in 1973. Zwick’s work raised the number of valid species to approximately 1800. Both the Zwick and Illies works were cited as the standard catalogs for four decades. Given some allowances made for species described since 1973, a resulting global estimate of approximately 2000 valid species names was referenced through much of the first decade of the 21st century [12].

A breakthrough achievement by Fochetti et al. [32], compiled a global, electronic checklist of valid names, synonyms, and distribution information. The resultant data were used by Fochetti and Tierno de Figueroa [33] to publish a global assessment of stonefly diversity, increasing the number of valid, extant species to 3497. The authors also tallied the number of extant species and genera for each biogeographic region and conducted a biogeographical analysis. The article summarized a wealth of other biological, distributional, and biogeographical information, making this one of the most important stonefly papers to date. The database of Fochetti et al. [32] was not intended to be a catalog and the data available from the website were limited to valid names and distribution information, but still provided data previously unavailable in digital form.

Simultaneously, Zwick had been compiling a comprehensive digital catalog that contained data from both the Illies [30] and Zwick [31] catalogs plus new data. About 15 years ago Zwick wished to hand over the responsibility for maintaining the database to others. In 2008, DeWalt became the curator of the database, and imported the data into a new biodiversity informatics platform called Species File Software [34].

The platform is maintained by several full-time biodiversity informatics programmers within the Species File Group (SFG) at the Illinois Natural History Survey. Species File Software captures nomenclatural data, literature references and citations, type information, occurrence data, images, sounds, links to other data, and distribution information mapped as shaded regions and as specimen point locations. Additional benefits of the platform include a user-friendly public interface, conformation to the International Code of Zoological Nomenclature [35], over 100 automated data integrity tests, biannual submission of data to Catalogue of Life (CoL) [36], and automated production of Darwin Core Archive formatted data for use by the Global Biodiversity Information Facility (GBIF) [17]. 

Distribution data in Species File Software utilizes the World Geographical Scheme for Recording Plant Distributions by Brummitt et al. [37]. Biodiversity Information Standards (TDWG) [38] employs a hierarchical format which provides the flexibility of conducting geospatial analyses at different scales. This scheme is composed of four nested levels of geography that reflect continental sized regions for Level 1, further subdivision for Level 2, and grading into political/administrative units at Levels 3 and 4. For example, a specimen from Ontario, Canada would be encoded as Level 1 “America, northern”, Level 2 “Eastern Canada”, Level 3 “Ontario”. Level 4 areas are available for other regions of the world. These distributions are represented as shaded maps produced using TDWG’s ArcGIS polygon shapefiles. Point data are displayed in a Google Maps platform. 

Plecoptera Species File (PSF) [9] is a global taxonomic database focusing on stonefly nomenclature, and reached 95% completion in 2010. It is currently the most cited stonefly taxonomic resource. It is the source of data for Plecoptera in the CoL and its taxonomic classification is used for stonefly data at GBIF, in the Barcode of Life Database [39], and in the Encyclopedia of Life [40]. The data are replicated throughout many other sources. Species and literature are continually added to the database. Publications in *Illiesia, The International Journal for Stonefly Research* [41] use a life science identifier (LSID) link for new taxa provided by PSF that resolves directly to a taxon page. The PSF is a mature resource and its data may now be used to conduct analyses on global and regional scales for taxonomic and geographic diversity and to relate the results to ecosystem services of stoneflies. 

The primary advantages of PSF over previous global Plecoptera taxonomic checklists [29,30,31,32] are the collaborative platform, longevity through endowment funding, adherence to biodiversity informatics standards, and a user-friendly interface. It is for these reasons that Plecoptera scientists have adopted its use and cite it frequently.

## 4. Global and Regional Species Richness

Queries were developed that identified all extant, valid species and the TDWG Level 1 regions in which they occur. A presence/absence data matrix was constructed for all species versus the following Level 4 regions: Europe, Africa (including Madagascar), Asia-Temperate (including China), Asia-Tropical, Australasia (Australia and New Zealand), North America (including Mexico), and South America (including Falkland Islands and Central America south of Mexico and Trinidad and Tobago). Parent taxa were removed from subspecies to avoid duplication. The number of families and species present were summarized for each of these continental regions.

## 5. Distribution, Diversity, and Classification

Data in PFS represent nearly 3000 references constituting 23,348 citations, each referencing a specific page number to a particular taxon. Currently, there are over 7000 specimen level records, about one-third being of primary types. The number of extant, valid species tallied in PSF stands at 3718 with 643 synonyms and homonyms (Table 1). We lack type specimen data for only 323 valid species, and this number is rapidly shrinking. PSF additionally includes 260 fossil species, many of which do not fit well into the classification of extant Plecoptera due to missing intermediate forms, unclear morphological features in known fossils, or an alternate classification system used by paleontologists.

**Figure 1:**
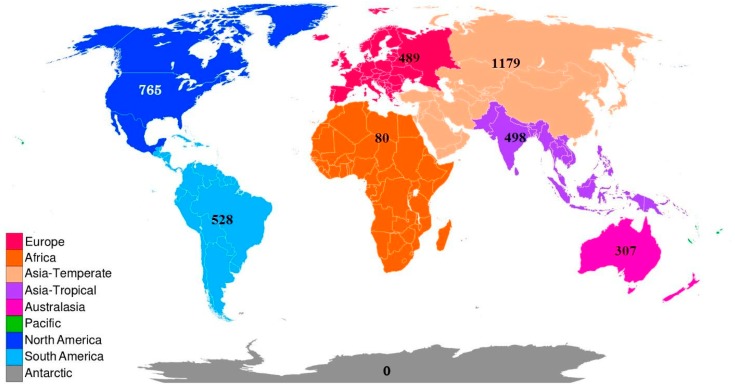
Biodiversity Information Standards (TDWG) Level 1 World Geographical Scheme [37] used in the Plecoptera Species File [9]. Numbers indicate known species richness through 2018.

**Figure 2:**
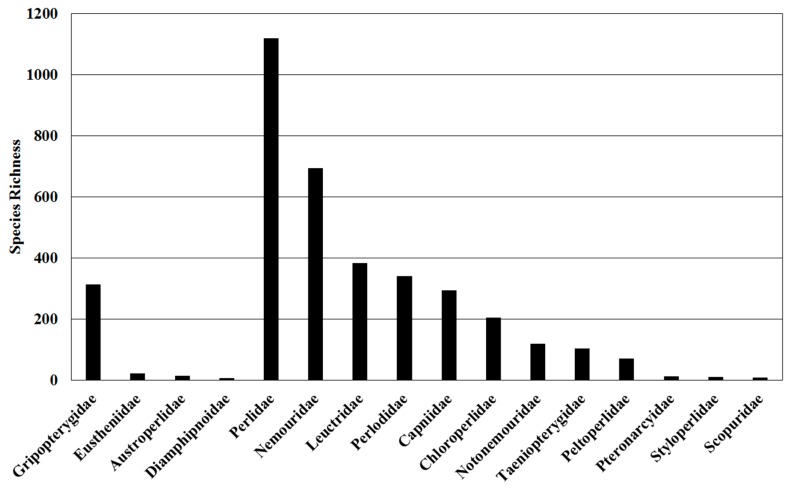
Species richness of Plecoptera families at the global scale.

The order Plecoptera contains two suborders, Arctoperlaria and Antarctoperlaria, which include 16 extant families (Table 2). Most species reside in the northern hemisphere in temperate regions, with the highest species richness occurring in the TDWG Level 1 region Asia-Temperate (Figure 1). Families vary greatly in species richness. The most diverse family, Perlidae, contains just over 1100 species (Table 1, Figure 2).

Arctoperlarian stoneflies mainly occur in the northern hemisphere (Table 2). They are composed of 12 families within two infraorders, the Euholognatha and Systellognatha. The infraorders roughly correspond to functional feeding groups of their nymphs: Euholognatha mostly feed on decayed plant matter, while the Systellognatha are primarily predatory. Some systellognathans (Peltoperlidae and Pteronarcyidae) feed on decayed plant matter. Two Arctoperlaria families (Perlidae and Notonemouridae) successfully colonized the southern hemisphere through two recent and independent natural invasions [42].

Antarctoperlarians are endemic to the southern hemisphere and include four families and 356 species in South America and Australasia (including New Zealand). The suborder is hypothesized to have originated on the Gondwana supercontinent [42] and diversified by allopatric speciation when the supercontinent drifted apart into South America, Africa, Antarctica, Australia, and India. Africa is one southern area that has surprisingly low species richness. No stoneflies are present in Antarctica, although historically it might have been a dispersal route among Gondwanan southern regions [42].

## 6. Species Diversity 

PSF includes 3718 valid stonefly species, which is 221 species more than Fochetti and Tierno de Figueroa [33]. This increase contrasts with the 435 species described since January 2008, reflecting the resolution of synonyms and some overlap in species counts between the two published works. Direct comparisons between various subdivisions of the world with the Fochetti and Tierno de Figueroa [33] results were challenging because their geographic data were not organized using the same hierarchical scheme. Some specific comparisons were possible for isolated land masses: We report 307 species for Australasia versus 295, confirming the low species richness in Australasia and a correspondingly slow rate of description. We found 80 species in Africa versus their 95. Several synonyms were resolved [43], and our methods dealing with subspecies largely account for the decrease in estimated species. Species diversity is low in Africa because the continent experienced much climate change over the past millennia and stoneflies are sensitive to changes in temperature and precipitation. Currently, Africa also has vast areas of arid land and warm equatorial rivers, conditions not conducive to high stonefly species richness.

Comparing large conjoined land masses proved to be more challenging. Fochetti and Tierno de Figueroa [33] reported 350 species for China, noting some uncertainty. We find that China has at least 615 species, 595 of which were described from China. It is unlikely that Fochetti and Tierno de Figueroa [33] had access to all the literature for China, and of course, the number of published papers has increased dramatically over the past decade. Chinese researchers now publish much of their work in English journals, significantly improving access to the literature. Editors of English journals have also requested that checklists come with Chinese stonefly papers which enhances our understanding of stonefly distribution in the country. As an indication of just how rapidly the Chinese fauna is being discovered and clarified, nearly 200 taxonomic papers have been published recently by the two most productive stonefly researchers in China, Yu-Zhou Du and co-authors (94 since 2001) and Weihai Li and co-authors (103 since 2004), most of them in English journals. A significant advance in our understanding of the Chinese fauna occurred with the publication of two new catalogs [44,45].

Our diversity measures for both North and South America have significantly increased over the previous estimates of 650 (PSF 765) and 474 (PSF 528) species, respectively. Some of the increase is attributable to newly described species. Another difference is that Mexico in the TDWG geospatial standard is part of North America, whereas in Fochetti and Tierno de Figueroa [33] it was not.

## 7. Rate of Species Description

Using the above data, we calculated the rate of description globally and across TDWG Level 1 regions. We also calculated a species description rate for China due to its perceived high rate. Species names were normalized to isolate year of description and the data sets sorted by year of description. To avoid overestimating species numbers, only species described within the region were retained in the checklist for that region. This necessitated examination of type data in the database, selective review of the literature, and the use of PSF distributional information to eliminate species that were described in a different region. Rates of description were calculated based on the number of species described between 1980 and 2018 (38 years). Species checklists for each region and country and state/province text distributions are available as supplemental data (Supplementary Data 1).

**Figure 3:**
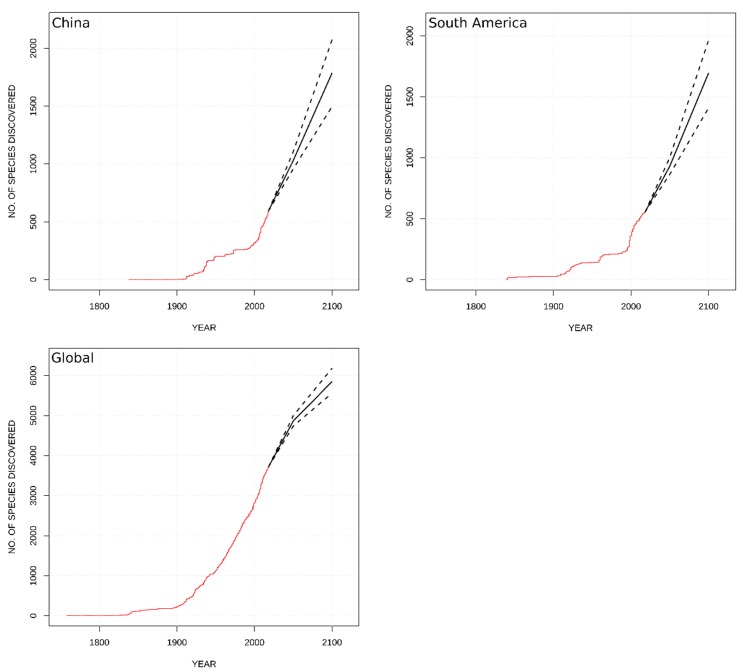
Observed species descriptions (**red**) with predicted species description curves (**black**) from the NHRP model for Global, China, and South America. Dashed lines are 95% confidence intervals.

The recent global rate of species description is 43.6 species/yr (Table 2) and shows no sign of decreasing (Figure 3). Among TDWG Level 1 regions, Asia-Temperate has the highest rate of species description, contributing 13.7 species/yr, over 31% of all annual species descriptions. China alone contributes 8.8 species/yr, 66% of the Asia-Temperate rate. South America also exhibits a high rate of description at 9.0 species/yr. Other TDWG Level 1 regions with moderate rates of description are Asia-Tropical and North America, both with >6 species/yr. The rates of description are considerably lower in both Europe and Australasia. Africa currently has the slowest rate of species description at approximately 0.4 species/yr. However, approximately 20 new species within the genus *Neoperla* Needham, 1905 (Perlidae) may result from Zwick’s anticipated revision of the genus [46].

## 8. Predicting Species Description in the 21st Century

Using the data developed in the rate of description, we examined species predicted to be described globally and for China and South America, two areas where researchers are contributing greatly to species description worldwide. Species discovery curves were fitted using a non-homogeneous renewal process (NHRP) model of Wilson and Costello 2005 [47] that accounts for variation in species descriptions caused by several factors (e.g., availability of researchers and funding, exploration of new geographical regions, and new species description techniques and species concepts). The NHRP model assumes that species descriptions follow a logistic-shaped curve with initially slow species description rates, followed by a period of rapid increase that gradually diminishes as new species become more difficult to find. The model follows this equation:

N/[1 + exp{−*v_1_*(*t − v_2_*)}], (1)

where *t* is the year, parameters *v_1_* and *v_2_* control the shape of the curve, and *N* is the expected number of species to be discovered [47,48]. In order to predict the remaining number of species to be discovered and quantify uncertainty in the prediction, the description years are sampled by the NHRP model as a random process following a logistic trend but with random variability included. The model predicted the number of new species descriptions from present to 2050 and 2100 based on the frequency distributions of past species descriptions.

Recent global rates of species description are high and not declining (Figure 3). Both China (Figure 3) and South America (Figure 3) also exhibit relatively high species description rates. For these three areas, it is difficult to predict when species description rates will plateau. Even for the global NHRP model, uncertainty is high for species description through the year 2100. The model estimated another 1400 ± 130–140 species described by 2050 (Table 3, Figure 3). The global model for the year 2100 predicted 2130 ± 290–340 new species described. China and South America are predicted to add 440 and 370 species by 2050, and 1190 and 1140 by 2100, respectively (Table 3, Figure 3).

Predicting future described species numbers based on past discovery curves can be inaccurate with incomplete datasets [49]. Surely, this is the case with stoneflies since rates of description are still high. However, at these rates of discovery, it is unlikely that even expert opinion could accurately predict future species description. Extrapolations from relative proportions of taxa in different geographical areas or habitats, or using gaps in body size distributions—under the assumption that larger, more conspicuous species are more likely to be discovered sooner—can also produce inaccurate predictions [48]. Species discovery predictions should become more accurate with time, so periodic recalculation of these estimates is warranted.

One of the most critical factors in reaching the projections of 2050 or 2100 is the number of researchers and funding available for species description. The number of laboratories where taxonomic work occurs is small, by our count, just under 30 (Table 4). Laboratories in South America and China each support several graduate students, and they are especially productive. One student, Zhi-Teng Chen, working in the Du laboratory published at least 18 papers in 2017 and 2018, all describing new species [9]. Similarly, students in South American laboratories are also describing many new species.

Conversely, laboratories in North America, Europe, and Australia/New Zealand are not training as many systematics-oriented students, partly because there are fewer undescribed species to be found. Change in emphasis at universities has also occurred, with fewer resources devoted to taxonomy and systematics. In these regions, students may be more successful if they pursue training in phylogenetics, evolution, biodiversity, and conservation of stoneflies.

Another factor that could help researchers meet the predictions in this paper is the expanded use of technology for species discovery and circumscription. The use of genetic markers, especially in conjunction with broad scale surveys of regions will speed up the process of finding new species [50]. Concerted campaigns such as that underway in Switzerland [51] can speed up the process of finding cryptic species, so long as taxonomists are involved in the formal description process [52]. 

## 9. Conclusions

Research in stoneflies is entering an exciting period of discovery. The majority of new species descriptions will likely occur in China, South America, and in Asia-Tropical. We also need much more work in India and the Himalayan Mountains. Although species description rates were found to be outpacing species extinction rates in a recent review [53], expediting species identification and circumscription of new species with molecular tools like DNA barcoding could be critical, especially if climate change increases extinction rates. Plecoptera species seem particularly vulnerable given their demonstrated regional imperilment. Sánchez-Bayoa and Wyckhuysmeet [16] suggest that 35% of species are in decline, 29% are in threatened status, and 19% are experiencing local or regional extinction within the areas studied (Europe and North America) with many species meeting IUCN criteria for inclusion in the Red List of Threatened Species.

Compiling all stonefly information already known to science is essential for efficient species descriptions, taxonomic revisions, predicting future species discoveries, ensuring conservation of species, synthesizing new hypotheses, and analyzing changes in functional traits and ecosystem services in time and space. The Plecoptera Species File has organized nearly all the available nomenclature details, literature, and distributional information for stoneflies into a single biodiversity informatics platform that is easily updatable and widely used by the stonefly research community. 

## Figures and Tables

**Table 1:** Families of Plecoptera and number of species within them globally and across major regions of the world through 2018 [9].

Classification	Global	Europe	Africa	Asia- Temperate	Asia-Tropical	Austral-Asia	North America	South America
Antarctoperlaria	-	-	-	-	-	-	-	-
Eusthenioidea	-	-	-	-	-	-	-	-
Diamphipnoidae	6	0	0	0	0	0	0	6
Eustheniidae	22	0	0	0	0	20	0	2
Gripopterygoidea	-	-	-	-	-	-	-	-
Austroperlidae	15	0	0	0	0	11	0	4
Gripopterygidae	313	0	0	0	0	214	0	99
Arctoperlaria	-	-	-	-	-	-	-	-
Euholognatha	-	-	-	-	-	-	-	-
Nemouroidea	-	-	-	-	-	-	-	-
Capniidae	295	28	4	104	9	0	167	0
Leuctridae	384	144	9	148	28	0	64	0
Nemouridae	694	150	13	338	148	0	80	0
Notonemouridae	120	0	39	0	0	62	0	20
Taeniopterygidae	104	48	2	27	1	0	36	0
Superfamily not assigned								
Scopuridae	8	0	0	8	0	0	0	0
Systellognatha	-	-	-	-	-	-	-	-
Perloidea	-	-	-	-	-	-	-	-
Chloroperlidae	204	23	1	71	6	0	107	0
Perlidae	1120	20	10	330	276	0	125	397
Perlodidae	340	76	2	118	6	0	152	0
Pteronarcyoidea	-	-	-	-	-	-	-	-
Peltoperlidae	71	0	0	24	23	0	24	0
Pteronarcyidae	12	0	0	2	0	0	10	0
Styloperlidae	10	0	0	9	1	0	0	0
	3718	489	80	1179	498	307	765	528

**Table 2:** Rate of stonefly species descriptions 1980 to 2018.

Area	Number Described	Rate of Description
Global	1657	43.6
Asia-Temperate	519	13.7
South America	343	9.0
China	335	8.8
Asia-Tropical	278	7.3
North America	250	6.6
Australasia	173	4.6
Europe	119	3.1
Africa	17	0.4

**Table 3:** The estimated number of species remaining to be discovered with a 95% probability of the estimate falling within the range provided in parentheses.

Location	Predicted Number of Species Discovered	
	2019–2050	2019–2100
Globally	1140 (1010–1280)	2130 (1840–2470)
China	440 (360–420)	1190 (900–1480)
South America	370 (310–450)	1140 (855–1400)

**Table 4:** Laboratories making recent contributions to Plecoptera species description. Affiliated workers and graduate students have been omitted due to space constraints.

Worker	Affiliation	Active in Regions
Pablo Pessacq	Centro de Investigaciones Esquel de Montaña y Estepa Patagónicos, Argentina	Argentina
Julia H. Mynott	La Trobe University, Australia	Australasia
Gunther Theischinger	New South Wales Department Planning & Environment, Australia	Australasia
Lucas S. Lecci	Universidade de São Paulo, Brazil	Brazil
Marcos Carneiro Novaes	Universidade Estadual Paulista, Brazil	Brazil
Tacio Duarte	Universidade Federal da Bahia, Brazil	Brazil
Pitágoras da Conseição Bispo	Universidade de São Paulo, Brazil	Brazil
Leandro Silva Barbosa	Museu Nacional, Brazil	Brazil
Gutierrez-Fonseca	University of Puerto Rico Rio Piedras	Central America
Weihai Li	Henan Institute of Science and Technology, China	China, SE Asia
Yu-Zhou Du	Yangzhou University, China	China, SE Asia
Maria del Carmen Zúñiga	Universidad del Valle, Colombia	Columbia
Gilles Vinçon	Grenoble, France	Europe
Wolfram Graf	University of Natural Resources and Applied Life Sciences, Austria	Europe
Tierno de Figueroa	University of Granada, Spain	Europe
Romolo Fochetti	University of Viterbo, Italy	Europe
Bill P. Stark	Mississippi College, USA	Global
Dávid Murányi	Hungarian Natural History Museum, Budapest	Global
Satoko Hanada	Fukuoka City, Japan	Japan
Takao Shimizu	Aqua Restoration Research Center, Japan	Japan, Taiwan
Scott A. Grubbs	Western Kentucky University, USA	North America
Boris C. Kondratieff	Colorado State University, USA	North America
Richard W. Baumann	Brigham Young University, USA	North America
R. Edward DeWalt	University of Illinois, USA	North America
Luke W. Myers	State University of New York, USA	North America
Valentina Teslenko	Russian Academy of Sciences, Vladivostok	Russian
Claudio G. Froehlich	University of São Paulo, Brazil	South America
Fernanda Avelino-Capestrata	Faculdades São José, Brazil	South America

## Supplementary Materials

The following are available online at https://www.mdpi.com/2075-4450/10/4/99/s1. Supplementary 1. DeWalt, R.E.; Ower, G.D. Global distribution of Plecoptera from Plecoptera Species File. Excel Spreadsheet.
